# Supplementary material for: Self-organization of Plk4 regulates symmetry breaking in centriole duplication
Source: Nat Commun. 2019 Apr 18;10:1810. doi: 10.1038/s41467-019-09847-x (PMC6472344; doi:10.1038/s41467-019-09847-x)
Supplement: Supplementary file 1 — Supplementary Information [file 41467_2019_9847_MOESM1_ESM.pdf]

Supplementary Information

Self-organization of Plk4 regulates symmetry breaking in centriole duplication

Yamamoto. S and Kitagawa. D

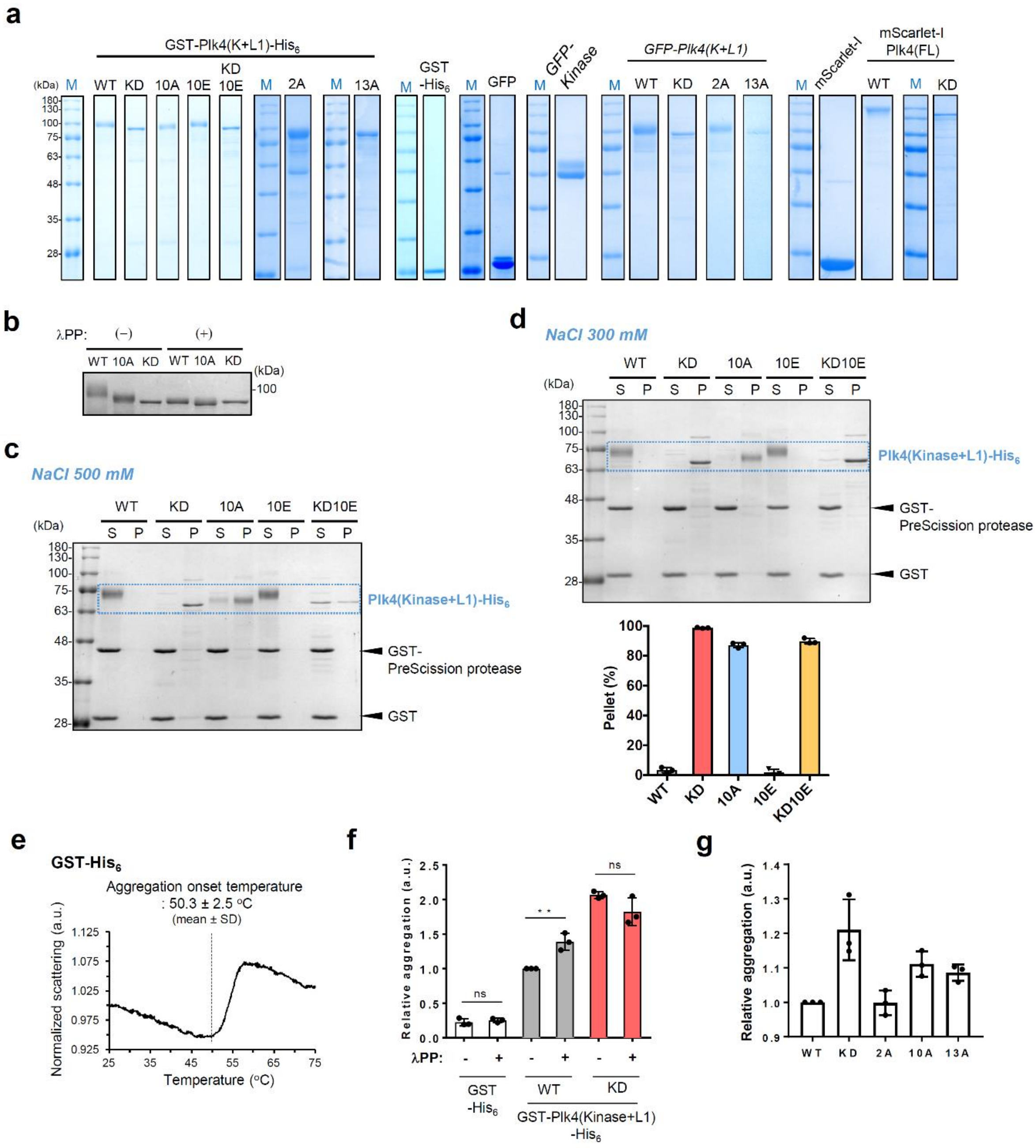

Supplementary Fig. 1. ***in vitro*** **analyses of purified Plk4. a** Purified proteins used in this study. Images show SDS-PAGE gel after CBB staining. Blue letters (M) indicate markers of molecular weight. **b** GST-Plk4(Kinase+L1) fragments were dephosphorylated with λPP. Image shows CBB-stained gel. Band shift of the WT was suppressed completely by the KD mutation and λPP treatment, and largely by 10A mutation. **c** Original image of spin-down assay in Fig. 1d (NaCl concentration, 500 mM). For (b) and (c), a dotted blue rectangle shows the bands of Plk4(Kinase+L1)-His$_6$. Black arrowheads indicate the positions of GST-tagged PreScission protease and cleaved GST-tag. Importantly, GST-PreScission protease and GST-tag were mostly detected in the soluble fraction. **d** Spin-down assay in lower NaCl concentration (300 mM). As in Fig. 1d and Supplementary Fig. 1c, 500 nM GST-Plk4 (Kinase+L1)-His$_6$ solutions were centrifuged and separated into supernatant (S) and pellet (P) fractions, after GST cleavage. Graph shows mean percentages of pellet and SD from three independent experiments. **e** Measurement of light scattering of GST-His$_6$ (100 μg/ml). Graph is a representative data of three experiments. Data was normalized to the scattering at 25°C. Dotted vertical line indicates aggregation onset temperature. **f** Proteostat aggregation assay with or without λPP. Fluorescence intensities were normalized to the WT λPP (-). Graph shows mean values and SD of three independent experiments. This experiment was performed at the same time of Fig. 1f and the data of λPP (-) are the same as Fig. 1f. **, $p<0.01$, ns, not significant (Two-tailed t-test). **g** Proteostat aggregation assay. Additional analysis of Plk4 mutants. Intensities were normalized to the WT. Graph shows mean values and SD of three independent experiments. Source data are provided as a Source Data file.

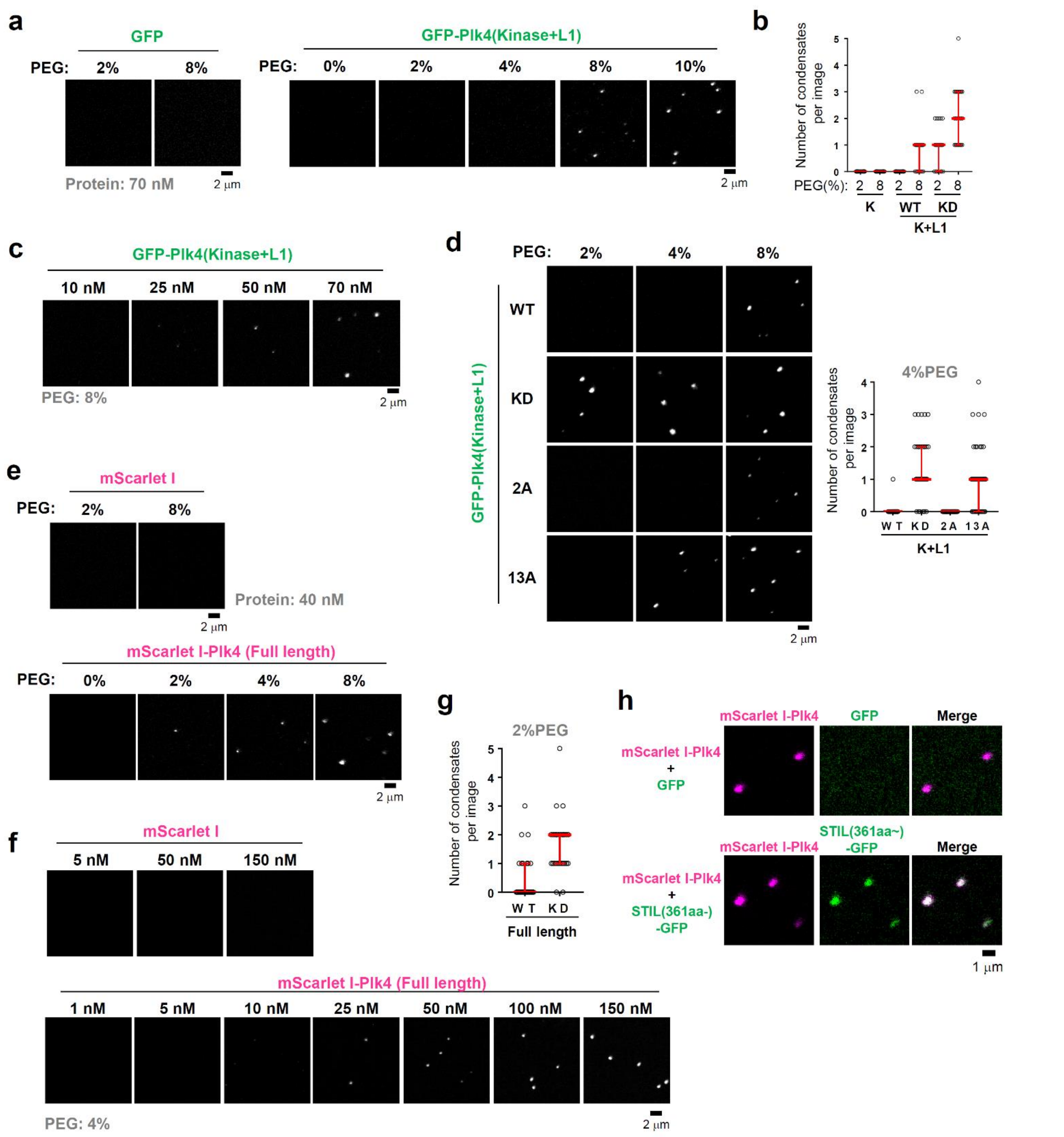

Supplementary Fig. 2. Characterization of condensation properties of fluorescence-labeled Plk4 *in vitro*. a Representative images of 70 nM GFP and GFP-Plk4 (Kinase+L1) at different PEG concentrations. Scale bar, 2 μm. **b** Quantification of condensate number per image (18.49 μm x18.49 μm) for Fig 2a. Red bars indicate median ± interquartile range (n = 24 images for each condition). K, GFP-Plk4 (Kinase); K+L1, GFP-Plk4 (Kinase+L1) **c** Representative images of GFP-Plk4 (Kinase+L1) at different protein concentrations in the presence of 8% PEG. Scale bar, 2 μm. **d** Representative images of 70 nM GFP-Plk4 (Kinase+L1) mutants at different PEG concentrations. Scale bar, 2 μm. Graph shows quantification of condensate number per image (18.49 × 18.49 μm) in a buffer containing 4% PEG. Red bars indicate median ± interquartile range (n = 48 images for each condition). **e** Representative images of 40 nM mScarlet I and mScarlet I-Plk4 (Full length) at different PEG concentrations. Scale bar, 2 μm. **f** Representative images of mScarlet I and mScarlet I-Plk4 (Full length) at different protein concentrations in the presence of 4% PEG. Scale bar, 2 μm. **g** Quantification of condensate number per image (18.49 × 18.49 μm) for Fig 2b. Condensation of 40 nM mScarlet I-tagged Plk4 (Full length) was induced in a buffer containing 2% PEG. Red bars indicate median ± interquartile range (n = 28 images for each condition). **h** 80 nM mScarlet I-Plk4 (Full length) was incubated in a buffer containing 4%PEG and then incubated

with 50 nM GFP or STIL(361-1287 a.a.)-GFP. A truncated form of STIL was used because full length STIL was difficult to be purified. Scale bar, 1 μm. Data including Fig.2a-b are representative of at least two independent experiments. Source data are provided as a Source Data file.

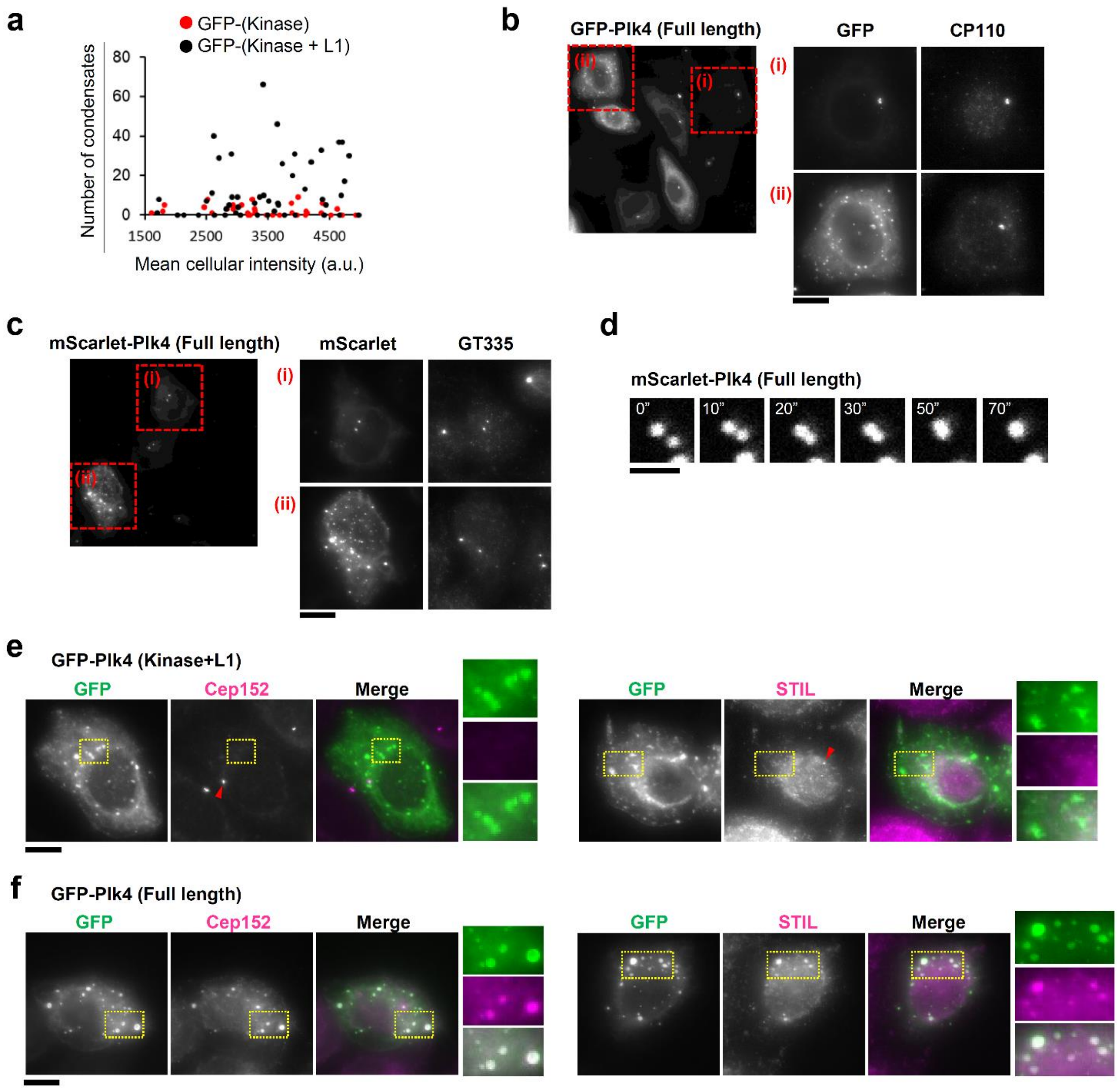

Supplementary Fig. 3. Characterization of Plk4 condensation in cells. a Related to Fig. 2c. Quantification of mean cellular intensity and the number of condensates in cells expressing GFP-Plk4 (kinase) or GFP-Plk4 (Kinase+L1) (n = 34 and 48 cells, respectively). Representative data of two independent experiments. Source data are provided as a Source Data file. **b, c** HeLa cells expressing GFP-Plk4 (Full length) (**b)** or mScarlet I-Plk4 (Full length) (**c**). Overexpression of Plk4 (Full length) also induced Plk4 condensation in cells. CP110 and GT335 were used as centriole markers. Scale bar, 10 µm. **d** Fusion event of mScarlet I-Plk4 (Full length)-induced cytoplasmic condensates. Scale bar, 2 µm. **e** HeLa cells expressing GFP-Plk4 (Kinase+L1) were stained with the indicated antibodies. Cep152 and STIL were undetectable in the Plk4 fragments-induced cytoplasmic condensates. Red arrowheads indicate centriolar signals. Scale bar, 10 µm. **f** HeLa cells expressing GFP-Plk4 (Full length) were stained with the indicated antibodies. Cep152 and STIL were frequently detected in the Plk4 (Full length)-induced cytoplasmic condensates, although it remains unknown whether Plk4 condensates concentrate Cep152 at centrioles in physiological condition. Scale bar, 10 µm.

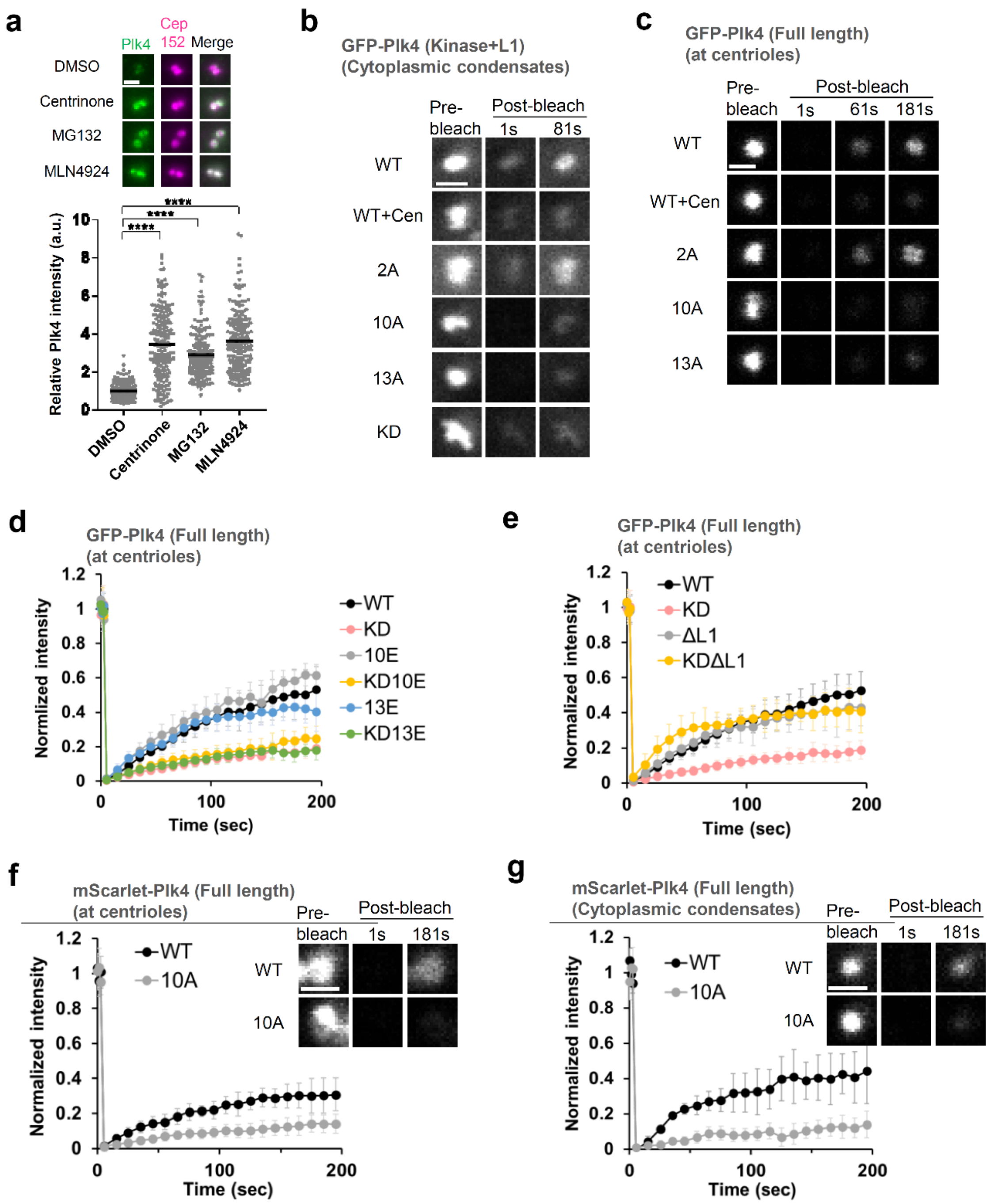

Supplementary Fig. 4. Supplemental data for characterization of Plk4 dynamics in cells. a Effects of inhibitors on endogenous centriolar Plk4 in HeLa cells. Cells were stained with the indicated antibodies and centriolar Plk4 intensity was quantified. Graph shows signal intensity of endogenous Plk4 at centrioles. Black bars show mean values (DMSO n = 189, Centrinone n = 196, MG132 n = 179, MLN4924 n = 195 centrioles) from three independent experiments. Data were normalized to the average intensity of DMSO. ****, $p < 0.0001$, one-way ANOVA. Scale bar, 1 µm. **b, c** Representative images of Fig.2h and 2i. Scale bar, 1 µm. **d, e** FRAP analysis of centriolar GFP-Plk4 (Full length) mutants in HeLa cells. Graph shows mean ± SD of (d)8 and (e)12 cells from at least two independent experiments. **d** The phospho-mimetic mutations did not seem to affect centriolar dynamics of the KD mutant. We assume that the phospho-mimetic mutations are insufficient to mimic the actual phosphorylation states of Plk4. **e** Centriolar dynamics of the KD mutant was recovered by deletion of the L1 domain, suggesting that the L1 domain contributes to autophosphorylation-regulated Plk4 condensation at centrioles. **f, g** FRAP analysis of centriolar mScarlet I-Plk4 (Full length) (**f**) and cytoplasmic mScarlet I-Plk4 (Full length) condensates (**g**) in HeLa cells. Autophosphorylation-regulated Plk4 dynamics were also observed in the case of mScarlet I-tagged Plk4. Graph shows mean ± SD of (f)10, (g)5 cells. Scale bar, 1 µm. Source data are provided as a Source Data file.

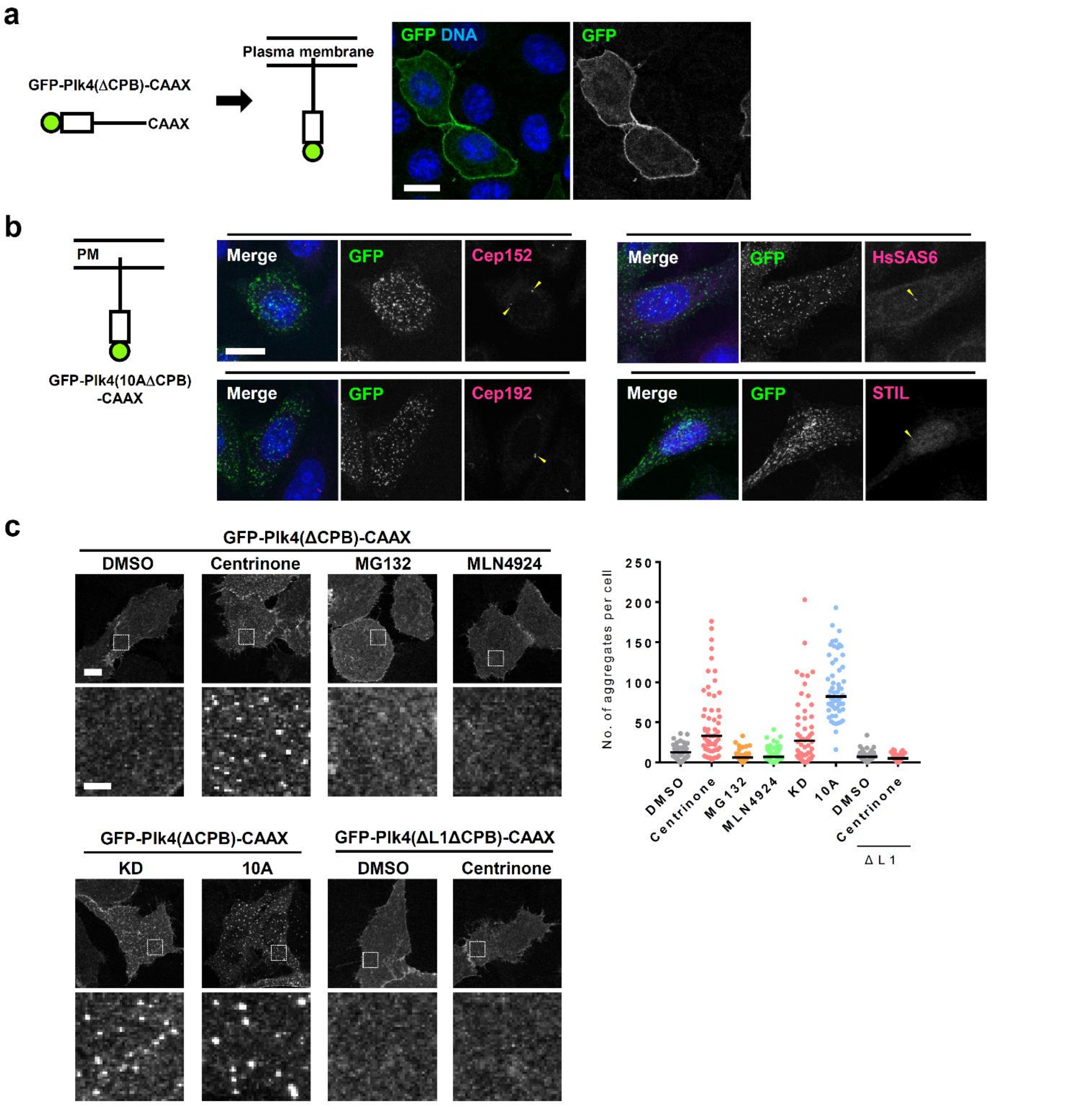

Supplementary Fig. 5. Plasma membrane tethering of Plk4 revealed differential condensation properties depending on its autophosphorylation state. a Image of HeLa cells expressing GFP-Plk4 (ΔCPB)-CAAX. GFP-Plk4 (ΔCPB)-CAAX was observed at the plasma membrane region. To exclude the effect of known Plk4 interactors, CPB domain that binds to Cep152 and Cep192 was deleted. Image was obtained by immunostaining with anti-GFP antibodies. Scale bar, 15 µm. **b** Immunostaining of Cep152, Cep192, HsSAS6 and STIL in HeLa cells expressing GFP-Plk4 (10AΔCPB)-CAAX. In this condition, each known interactor was undetectable at the foci of membrane-tethered Plk4. Yellow arrowheads indicate centrosomal signal of the indicated proteins. Scale bar, 15 µm. **c** GFP-Plk4 (ΔCPB)-CAAX distribution at the plasma membrane region of HeLa cells stained with anti-GFP antibodies. Lower panels are magnified images of the indicated region of each cell. Cells were treated with DMSO, 100 nM centrinone (20 hours), 10 µM MG132 (5 hours) or 10µM MLN4924 (5 hours) and fixed 20 hours after plasmid transfection. GFP-Plk4 (ΔCPB)-CAAX uniformly localized to the plasma membrane. In stark contrast, inhibition of the Plk4 kinase activity with centrinone or overexpression of the KD and 10A mutants, significantly induced condensation of membrane-localized Plk4. Such condensation was unlikely to be because of inhibition of autophosphorylation-dependent protein degradation, because inhibition of the degradation pathway by treatment with MG132 or MLN4924 did not influence the uniform localization of membrane-localized Plk4. Deletion of L1 (ΔL1) suppressed centrinone-induced condensation, suggesting that L1 domain is required for the condensation

of Plk4. Graph shows number of condensates per plasma membrane region with median (black bar) of (DMSO n = 60, Centrinone n = 58, MG132 n = 61, MLN4924 n = 61, KD n = 54, 10A n = 58, ΔL1+DMSO n = 53, ΔL1+Centrinone n = 55 cells) from two independent experiments. Scale bar, 10 μm; 2 μm in magnified images. Source data are provided as a Source Data file.

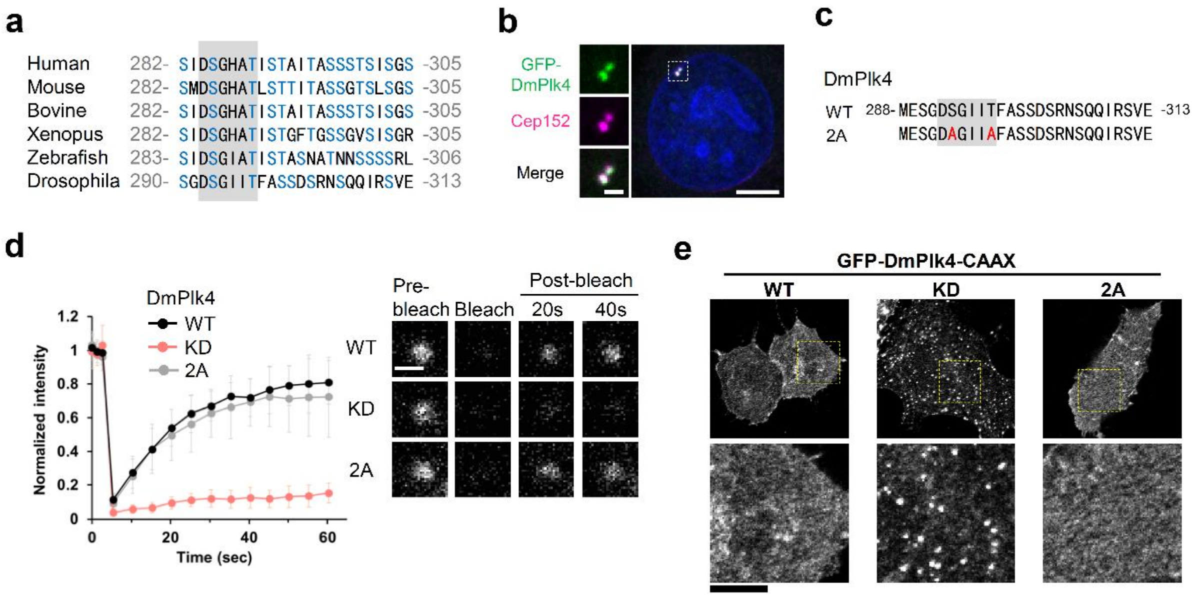

Supplementary Fig. 6. Evolutional conservation of Plk4 properties. **a** Conservation of S/T enriched sequence of Plk4. Amino acid sequence alignment of Plk4s is shown. Gray background indicates degron motif. Serine and threonine are shown with blue letters. S/T enriched nature around degron motif seems to be conserved across species. **b** HeLa cells expressing GFP tagged *Drosophila* Plk4 (DmPlk4). After transfection of the plasmid encoding GFP-DmPlk4, cells were fixed and stained with anti-GFP and Cep152 antibodies. Scale bar, 5 µm in larger image and 1 µm in magnified image. **c** Amino acid sequence of degron motif and the neighboring region in DmPlk4. Red letters, mutation sites; Gray background, degron motif. **d** FRAP analysis of GFP-DmPlk4 overexpressing in HeLa cells. The KD mutation (D156A), but not 2A (in degron) suppressed DmPlk4 dynamics, implying that the centriolar dynamics of DmPlk4 are also regulated by its kinase activity rather than by its degradation. Intensities were normalized with the average of three pre-bleach signals. Graph shows mean values and SD (n = 15 cells for each condition) from three independent experiments. Scale bar, 1 µm. Source data are provided as a Source Data file. **e** GFP-DmPlk4 (Full length)-CAAX distribution at the plasma membrane region of HeLa cells stained with anti-GFP antibodies. Lower panels are magnified images of the indicated region of each cell. DmPlk4 showed large number of condensates on the plasma membrane in its KD form, but not in the WT and 2A form, showing similar property with human Plk4 (Supplementary Fig. 5). Scale bar, 5 µm in magnified images.

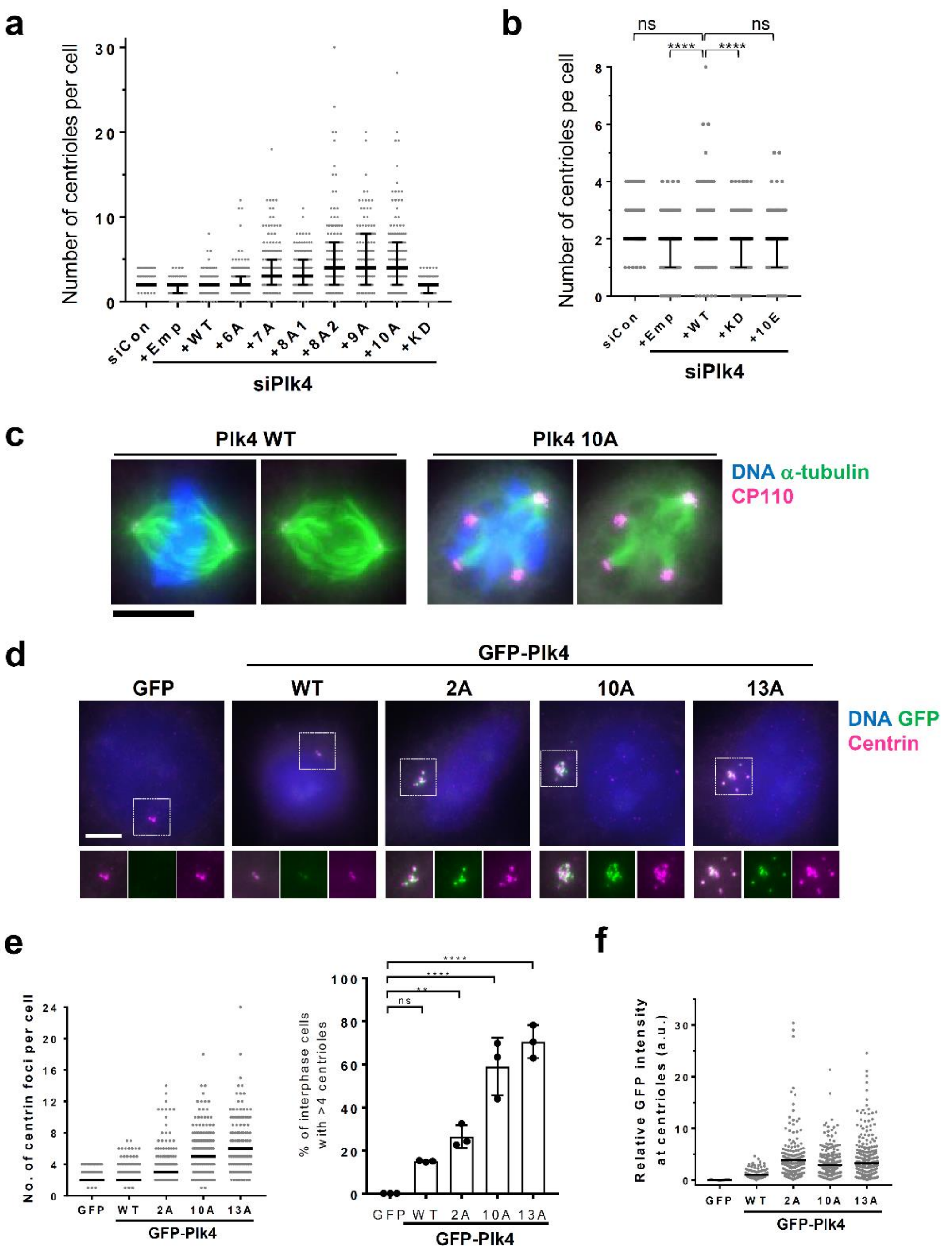

Supplementary Fig. 7. Centriole overduplication assay. Supplemental data for Fig. 3. a, b Centriole overduplication assay, related to Fig. 3d. The number of CP110 foci was counted in the same experiments with Fig. 3d. Black bars show median ± interquartile range (siControl n = 146, siPlk4+Emp n = 159, siPlk4+WT n = 153, siPlk4+6A n = 153, siPlk4+7A n = 166, siPlk4+8A1 n = 155, siPlk4+8A2 n = 148, siPlk4+9A n = 151, siPlk4+10A n = 157, siPlk4+KD n = 151 cells) from three independent experiments. **b** The phospho-mimetic mutation (10E) (n = 168 cells) did not seem to show significant phenotype. Black bars show median ± interquartile range. ****, $p < 0.0001$, ns, not significant, Kruskal-Wallis test. **c** Representative images of mitotic cells expressing Plk4 WT or 10A tagged with 3xFLAG under the CMV mutant promoter. HeLa cells were fixed 48 hours after plasmid transfection and immunostained with α-tubulin and CP110. In merged images, green, magenta and blue represent α-tubulin, CP110 and DNA, respectively. Scale bar, 5 μm. Over-amplified centrioles induced by expression of Plk4 10A associate with spindle microtubules, suggesting that over-amplified centrioles function as MTOCs in mitosis. **d-f** Centriole over-duplication assay. GFP-Plk4 was exogenously expressed in HeLa cells under human *Plk4* promoter. Cells were fixed 48 hours after plasmid transfection and stained with the indicated antibodies. In this condition, expression of GFP-Plk4 WT resulted in minimal centriole overduplication. However, the 2A mutation in the degron motif of Plk4 led to significant centriole overduplication, presumably by increasing total expression level of Plk4. Importantly, 10A and 13A mutations induced over-duplication of centrioles, to a greater extent than the WT and the

2A mutation. **d** Representative images. Scale bar, 5 μm. **e** Left graph represents the number of Centrin foci in GFP-positive interphase cells. Black bars show median (GFP n = 147, WT n = 114, 2A n = 152, 10A n = 153, 13A n = 135 cells) from three independent experiments. Right graph represents the percentages of interphase cells which have >4 centrin foci. Graph represents mean percentages and SD of three independent experiments. **, $p < 0.01$, ****, $p < 0.0001$, ns, not significant, one-way ANOVA. **f** Graph represents GFP signal intensity at centrioles. Data were normalized to the average intensity of the WT. Black bar shows mean signal intensity (GFP n = 177, WT n = 174, 2A n = 228, 10A n = 313, 13A n = 359 centrioles) from two independent experiments. Source data are provided as a Source Data file.

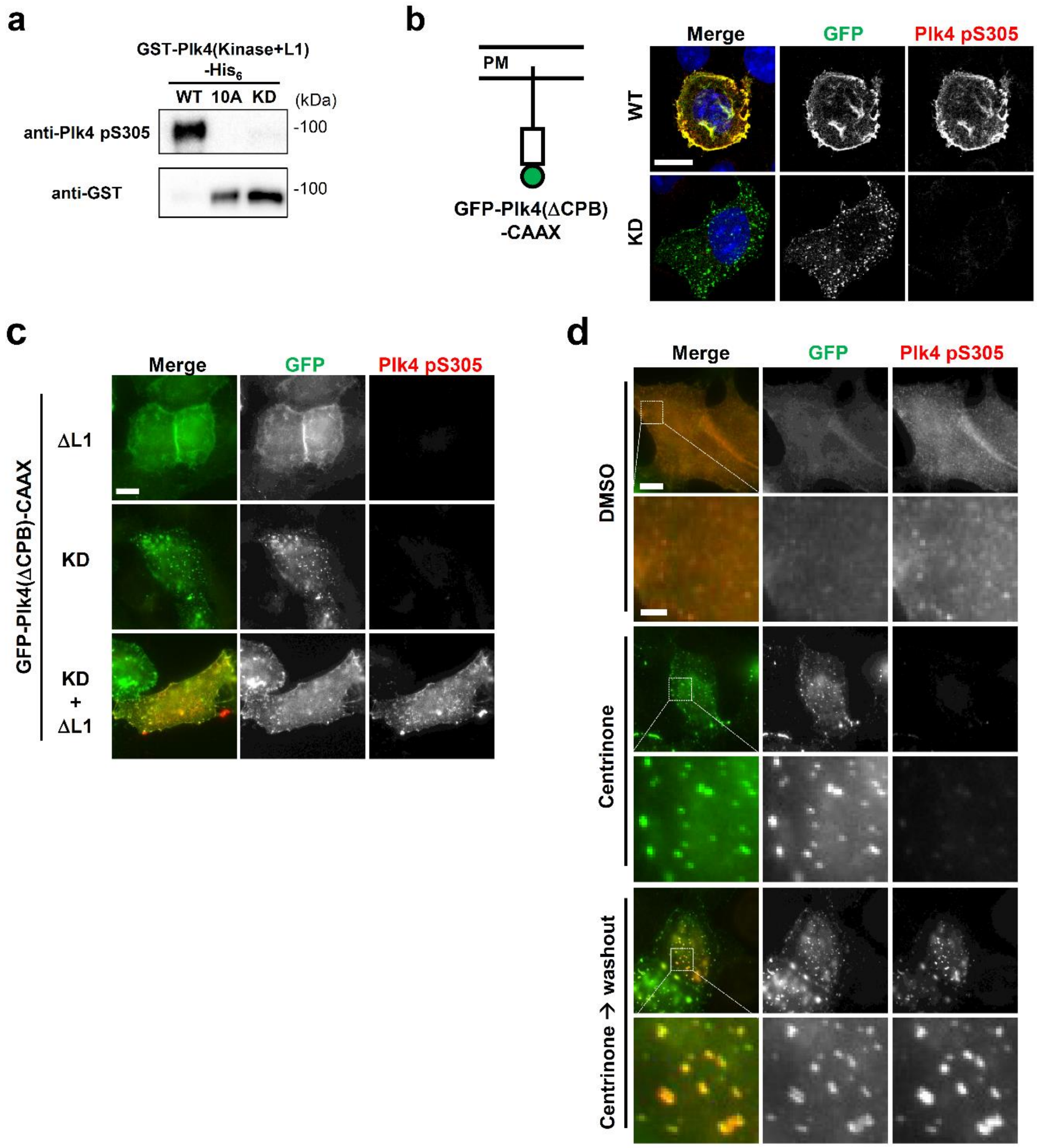

Supplementary Fig. 8. Validation of the specificity of anti-Plk4pS305 antibodies. a Immunoblotting of GST-Plk4(kinase+L1)-His_6 with anti-Plk4pS305 (upper) and anti- GST (lower) antibodies. Anti-pS305 antibodies specifically reacted with purified Plk4 WT fragments, but hardly with 10A and KD. Because the band of WT was smeared probably by phosphorylation, detection of the WT with anti-GST antibodies became weaker, compared with the KD. Source data are provided as a Source Data file. **b, c** Immunostaining of Plk4pS305 in HeLa cells expressing GFP-Plk4 (ΔCPB)-CAAX. Cells were fixed and stained with the indicated antibodies. Anti-Plk4pS305 antibodies specifically reacted with membrane-tethered Plk4 (WT), but hardly with KD. Scale bar, 15 μm (**b**) and 10 μm (**c**). **d** Cells expressing GFP-Plk4 (ΔCPB)-CAAX were treated with 100 nM Centrinone (20 hours) and then released from centrinone (1 hour). Cells were fixed and stained with the indicated antibodies. Interestingly, centrinone-induced condensates were not dissociated even in the re-activation of Plk4 upon centrinone washout, suggesting that inactivation of Plk4 led to irreversible aggregation state of Plk4. Scale bar, 10 μm.

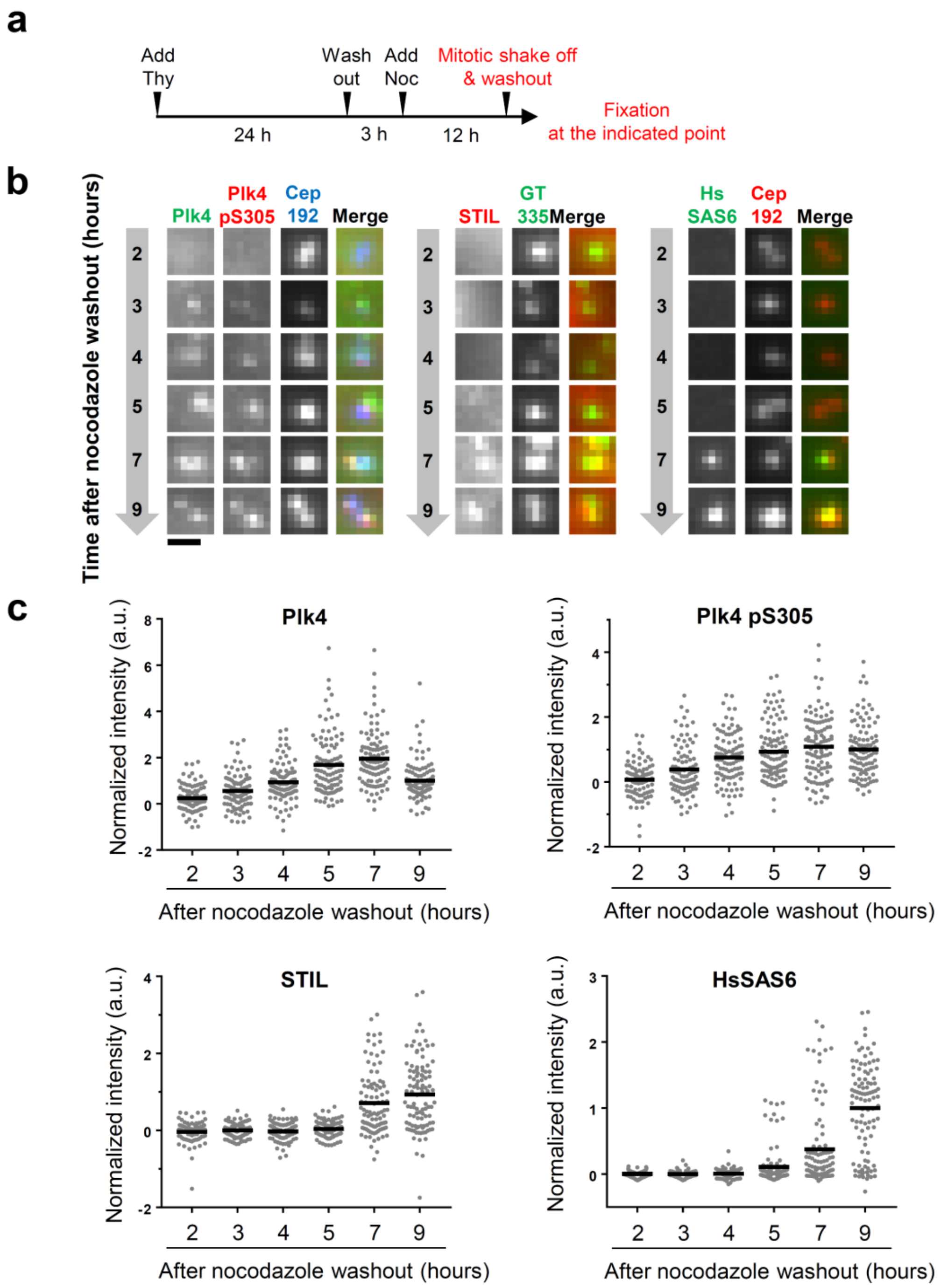

Supplementary Fig. 9. Monitoring Plk4, Plk4pS305, STIL and HsSAS6 through G1 phase progression. a Scheme of the experimental condition, related to Fig.4c. HeLa cells were treated with 100 µM Thymidine for 24 hours and then released for 3 hours. Cells were then treated with 50 ng/ml nocodazole for 12 hours. Mitotic cells were collected by mitotic shake-off and released from nocodazole by washout. Cells were then seeded onto coverslips and fixed at the indicated time point. **b** Representative images of centriolar Plk4, Plk4pS305, STIL and HsSAS6. Scale bar, 1 µm. **c** Quantification data of centriolar Plk4, Plk4pS305, STIL and HsSAS6 intensities. Data were same as Fig. 4c. Intensities were normalized to the average intensity at 9 hours after nocodazole washout. Black bars show mean values of centriolar intensities (For each time point (2, 3, 4, 5, 7 and 9 hours), Plk4 n = 94, 92, 99, 106, 108 and 102 centrioles, Plk4pS305 n = 94, 92, 99, 106, 108 and 102 centrioles, STIL n = 72, 91, 97, 92, 98 and 103 centrioles, HsSAS6 n = 99, 100, 102, 107, 103 and 102 centrioles) from two independent experiments. Source data are provided as a Source Data file.

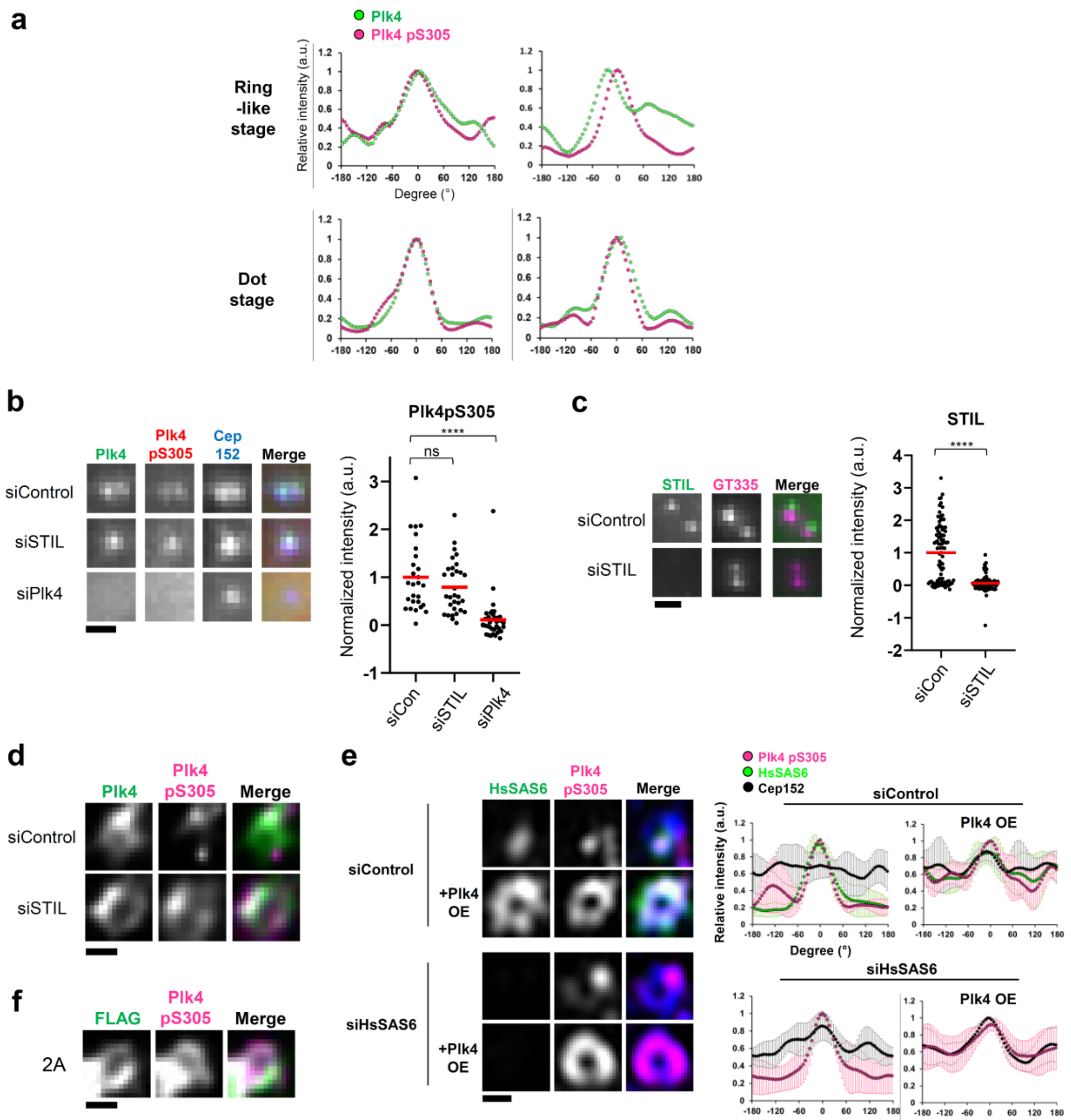

Supplementary Fig. 10. A single focus of autophosphorylated Plk4 is generated independently of STIL-HsSAS6 loading. a Relative intensities of Plk4 (Green) and Plk4pS305 (Magenta) in Fig. 4b were quantified along Cep152 ring and maximum peaks of Plk4pS305 were set to 0 °. Intensities were normalized to each maximum intensity. **b - d** Cells were treated with the indicated siRNA for 48 hours and arrested at G1 phase with 10 μM Lovastatin. Cells were then stained with the indicated antibodies. Red bars show mean values of centriolar intensities ((**b**) Plk4pS305 siControl n = 27, siSTIL n= 31, siPlk4 n = 39 centrioles and (**c**) STIL siControl n = 89, siSTIL n = 89 centrioles). Scale bar, 1 μm (**b** and **c**). ****$p < 0.0001$, ns, not significant, (b) one-way ANOVA and (c) two-tailed t-test. **d** Representative images of centriolar Plk4 and Plk4pS305. Images were obtained by TCS SP8 HSR system with deconvolution. Scale bar, 0.3 μm. **e** Plk4pS305 patterns in S-phase arrested HeLa cells. Cells were treated with siRNA for 48 hours and 6 μM aphidicolin for 24 hours. For Plk4 overexpression (OE), cells were transfected with plasmids encoding Plk4-3 × FLAG under CMV promoter and fixed 24 hours after plasmid transfection. Relative intensities of each protein were quantified along Cep152 ring and maximum peaks of Plk4pS305 intensity were set to 0 °. Intensities were normalized to each maximum intensity. Graph shows mean intensities ± SD (siControl n = 5, siControl+Plk4OE n = 5, siHsSAS6 n = 6, siHsSAS6+Plk4OE n = 8 centrioles) from two independent experiments. Scale bar, 0.3 μm. **f** Representative images of centriolar Plk4 and Plk4pS305 in cells expressing Plk4-3xFLAG 2A under the CMV mutant promoter. Cells were transfected with siPlk4 and plasmid and arrested at G1 phase with Lovastatin. Images were obtained by TCS SP8 HSR system with deconvolution. Scale bar, 0.3 μm. Source data are provided as a Source Data file.

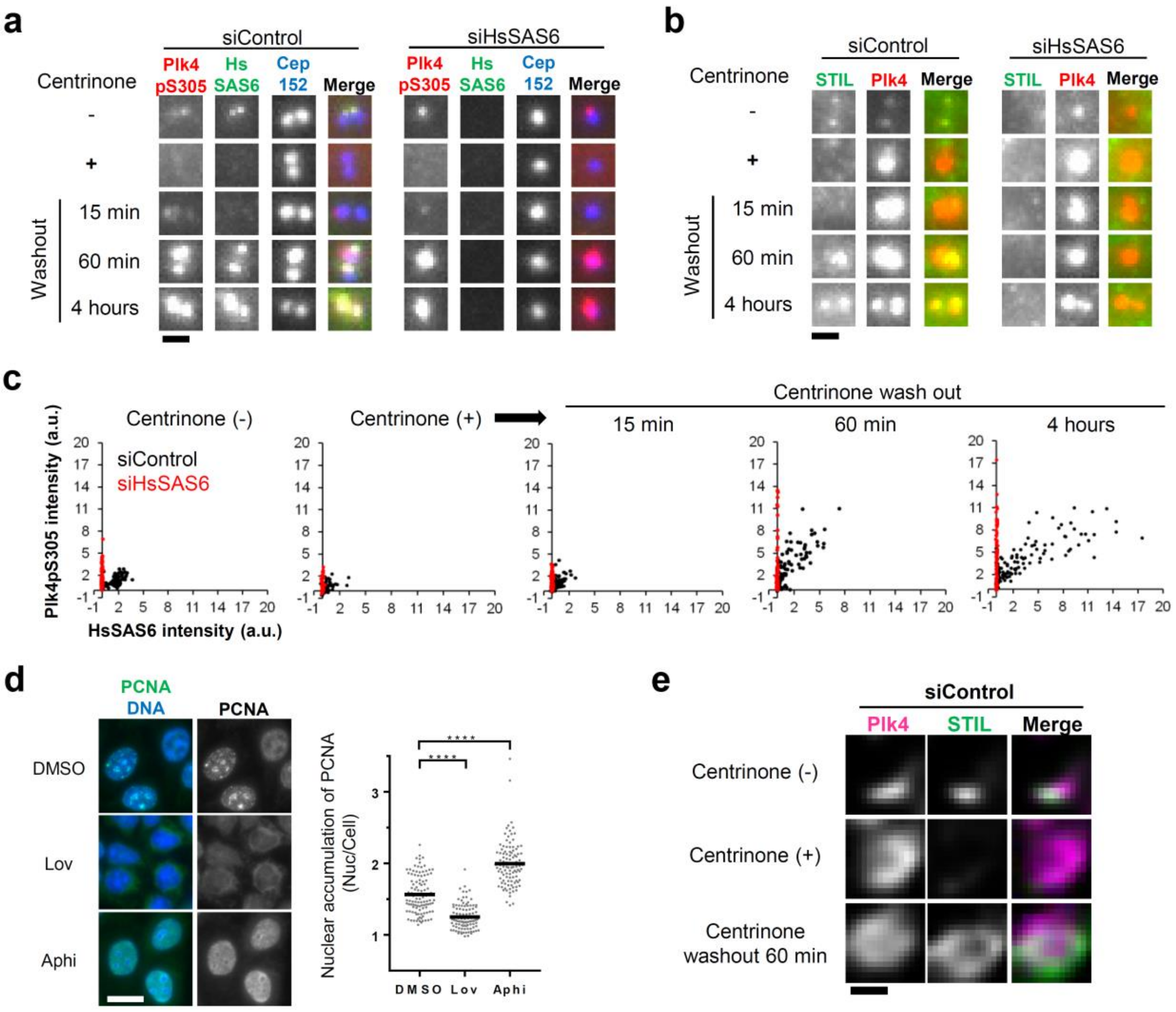

Supplementary Fig. 11. Supplemental data for Fig. 5. a, b Related to Fig5b and c, Representative merged images of immunostained HsSAS6, Plk4pS305, STIL and Plk4 in the indicated condition. For **a**, Cep152 was used as a centriole marker. Scale bar, 1 μm. **c** Correlation between HsSAS6 and Plk4pS305 intensity. Data were the same as Fig. 5b and 5c. Black and red dots represent the data from siControl and siHsSAS6-treated cells, respectively. **d** Validation of cell cycle synchronization with chemicals by immunostaining against PCNA. Cells were treated with 6 μM aphidicolin (Aphi, 24 hours) or 10 μM Lovastatin (Lov, 20 hours). Nuclear accumulation of PCNA was calculated from mean nuclear intensity divided by mean cellular intensity. Compared to DMSO, Lovastatin suppressed nuclear accumulation of PCNA and aphidicolin increased nuclear PCNA in most cells, suggesting cell cycle synchronization in G1 and S phase, respectively. Graph shows mean value (black bar) (DMSO n = 109, Lov n = 102, Aphi n = 104 cells). $^{**}p < 0.01$, $^{****}p < 0.0001$, ns, not significant, one-way ANOVA. Scale bar, 20 μm. Source data are provided as a Source Data file. **e** Magnified images of centrioles immunostained with Plk4 and STIL in the indicated condition, related to Fig. 5. Images were obtained by TCS SP8 HSR system with deconvolution. Scale bar, 0.3 μm.
