# Supplementary material for: Self-organization of Plk4 regulates symmetry breaking in centriole duplication
Source: Nat Commun. 2019 Apr 18;10:1810. doi: 10.1038/s41467-019-09847-x (PMC6472344; doi:10.1038/s41467-019-09847-x)
Supplement: Supplementary file 3 — Reporting Summary [file 41467_2019_9847_MOESM3_ESM.pdf]

natureresearch

Corresponding author(s): Daiju Kitagawa

Reporting Summary

Nature Research wishes to improve the reproducibility of the work that we publish. This form provides structure for consistency and transparency in reporting. For further information on Nature Research policies, see Authors & Referees and the Editorial Policy Checklist.

Statistical parameters

When statistical analyses are reported, confirm that the following items are present in the relevant location (e.g. figure legend, table legend, main text, or Methods section).

n/a	Confirmed	
☐	☒	The exact sample size (*n*) for each experimental group/condition, given as a discrete number and unit of measurement
☐	☒	An indication of whether measurements were taken from distinct samples or whether the same sample was measured repeatedly
☐	☒	The statistical test(s) used AND whether they are one- or two-sided<br>*Only common tests should be described solely by name; describe more complex techniques in the Methods section.*
☐	☒	A description of all covariates tested
☐	☒	A description of any assumptions or corrections, such as tests of normality and adjustment for multiple comparisons
☐	☒	A full description of the statistics including central tendency (e.g. means) or other basic estimates (e.g. regression coefficient) AND variation (e.g. standard deviation) or associated estimates of uncertainty (e.g. confidence intervals)
☐	☒	For null hypothesis testing, the test statistic (e.g. *F*, *t*, *r*) with confidence intervals, effect sizes, degrees of freedom and *P* value noted<br>*Give P values as exact values whenever suitable.*
☒	☐	For Bayesian analysis, information on the choice of priors and Markov chain Monte Carlo settings
☒	☐	For hierarchical and complex designs, identification of the appropriate level for tests and full reporting of outcomes
☒	☐	Estimates of effect sizes (e.g. Cohen's *d*, Pearson's *r*), indicating how they were calculated
☐	☒	Clearly defined error bars<br>*State explicitly what error bars represent (e.g. SD, SE, CI)*

Our web collection on statistics for biologists may be useful.

Software and code

Policy information about availability of computer code

Data collection	Images were collected using SoftWoRx (Applied Precision), LAS X (Leica), ZEN (Zeiss) and NIS-Element AR software (Nikon). Fluorescence intensity of purified proteins were obtained using Filter Max F5 with SoftMax software or FLUOstar OPTIMA (BMG).
Data analysis	Aggregation onset temperature of purified proteins was analyzed using the Prometheus PR. ThermControl software (NanoTemper technologies). 3D-SIM image reconstruction were performed with NIS-Element AR software. Quantification of signal intensities was performed using Fiji or Image J. Deconvolution of confocal images was perfomed using Huygens essential software (SVI). Statistical analysis was performed using GraphPad Prism7 and 8.

For manuscripts utilizing custom algorithms or software that are central to the research but not yet described in published literature, software must be made available to editors/reviewers upon request. We strongly encourage code deposition in a community repository (e.g. GitHub). See the Nature Research guidelines for submitting code & software for further information.

Data

Policy information about availability of data

All manuscripts must include a data availability statement. This statement should provide the following information, where applicable:

- Accession codes, unique identifiers, or web links for publicly available datasets
- A list of figures that have associated raw data
- A description of any restrictions on data availability

The data that support the findings of this study are available from the corresponding author upon request.

Field-specific reporting

Please select the best fit for your research. If you are not sure, read the appropriate sections before making your selection.

☒ Life sciences ☐ Behavioural & social sciences ☐ Ecological, evolutionary & environmental sciences

For a reference copy of the document with all sections, see nature.com/authors/policies/ReportingSummary-flat.pdf

Life sciences study design

All studies must disclose on these points even when the disclosure is negative.

Sample size	No statistical method was used to predetermine sample size.
Data exclusions	No data were excluded from the analyses.
Replication	Reproducibility was confirmed. The number of experiments is described in the figure legends.
Randomization	For imaging experiments, we assessed aggregates and cells from several fields for each experiment. The field were chosen randomly. Once a field was determined, we counted all cells which match the criteria within the filed.
Blinding	The investigators were not blinded to the sample ID during experiments and outcome assessment because the cells in randomly selected area were analyzed objectively.

Reporting for specific materials, systems and methods

Materials & experimental systems

n/a	Involved in the study
☐	☒ Unique biological materials
☐	☒ Antibodies
☐	☒ Eukaryotic cell lines
☒	☐ Palaeontology
☒	☐ Animals and other organisms
☒	☐ Human research participants

Methods

n/a	Involved in the study
☒	☐ ChIP-seq
☒	☐ Flow cytometry
☒	☐ MRI-based neuroimaging

Unique biological materials

Policy information about availability of materials

Obtaining unique materials	anti-Plk4pS305 antibody was obtained by immunizing a rabbit with a S305-phosphorylated peptide.

Antibodies

Antibodies used	Detailed information about the antibodies used in this study are described in "Methods" section.
Validation	Cep152 (Bethyl laboratories, A302–480A): The specificity was validated on the manufacturer's website and a literature (Hatch et al., 2010)., Cep192 (Bethyl laboratories, A302–324A): The specificity was validated on the manufacturer's website and literatures., STIL (Abcam, ab89314): The specificity was validated on the manufacturer's website, literatures and in this

manuscript (Supplementary Fig. 10c)., CP110 (Proteintech, 12780-1-AP): The specificity was validated on the manufacturer's website and literatures., GFP (MBL, 598): The specificity was validated on the manufacturer's website and literatures., Plk4 phospho-S305: The specificity was validated in this manuscript (Supplementary Fig. 8 and 10b)., Plk4 (Merck Millipore, clone 6H5, MABC544): The specificity was validated on the manufacturer's website., HsSAS6 (Santa cruz Bio-technology, Inc., sc-81431): The specificity was validated in this manuscript (Figure 5b)., Centrin-2 (Merck Millipore, clone 20H5, 04-1624): The specificity was validated on the manufacturer's website and literatures., PCNA (Santa cruz Bio-technology, Inc., sc-56): The specificity was validated on the manufacturer's website and literatures., GFP (Invitrogen, A11120): The specificity was validated on the manufacturer's website and literatures., GST (MBL, M071-3): The specificity was validated on the manufacturer's website and literatures. FLAG (Sigma, F1804): The specificity was validated on the manufacturer's website and literatures. a-tubulin (sigma, T5168): The specificity was validated on the manufacturer's website and literatures. Polyglutamylation Modification (GT335) (AdipoGen, AG-20B-0020-C100): The specificity was validated on the manufacturer's website and literatures.

Eukaryotic cell lines

Policy information about cell lines

Cell line source(s)	HeLa cells were obtained from ECACC.
Authentication	HeLa have been authenticated by STR profiling in ECACC.
Mycoplasma contamination	It have been confirmed that HeLa were not contaminated with mycoplasma by indirect DNA stain using Hoechst 33258 with indicator cells (Vero cells).
Commonly misidentified lines (See ICLAC register)	No commonly misidentified cell lines were used.
